# 

**Published:** 1888-01

**Authors:** 


### INDEX.

A.

Abdominal Surgery ... 307
Abscess Acute Psoas, Dr. J. M. F. Gaston ... 282
Abscess, perinephritic, Dr. G. G. Roy ... 242
Abscess, renal, Dr. Arch Dixson ... 1
Academy of Medicine, Baltimore, Dr. J. J. Chisholm ... 287
Acetanilid ... 141, 390
Acetanilid in enteric fever ... 141
Acetic acid and ergot in post partum hemorrhage ... 145
Acid boracic, use of the ... 429
Acid hydrochloric and cocaine ... 274
Acid hydrochloric in dyspepsia ... 234
Acid in diarrhœa of children. Lactic ... 230
Acid, indigestion ... 307
Acid treatment in intestinal diseases of children ... 185
Acidi muriatici mistura ... 273
Acne, treatment of ... 35
Aconitia, dose of. A warning ... 428
Address of Judge Nesbit ... 126
Advertisements in religious newspapers. Quack ... 116
Advertisers, our ... 37
Agalactic, antipyrine as an ... 430
Agaricin ... 194, 300
Agent, a new therapeutic ... 315
Agnew, death of Dr. C. R. ... 235
Ague ... 28, 187
Ague cake ... 187
Air inhalations for phthisis ... 462
Air for operating switches, compressed ... 312
Alabama, medical association of ... 235
Alcholism, a hyponitic for ... 313
Alcoholism, strychnine in ... 309
Alum in typhoid fever ... 216
Amblyopia, tobacco ... 65
American Association for the development of science ... 391
American association of obstetricians and gynecologists ... 315
American exhibit at the Paris exposition ... 436
American medical association ... 76, 158, 213, 315 ... 316
American Rhinological Association ... 276
Ammonia in asthma, iodide of ... 256
Ammonia, muriate of ... 74, 273
Ammonium in uric acid calculi, biborate of ... 139
Amnesia. a remarkable case of ... 32
Amyl nitrite in opium poisoning ... 180
Amylene, hydrate of ... 66, 183
Anœsthesia, dangers of Dr. G. G. Roy ... 241
Anœsthesia, obstetric, Dr. N. M. Dodson ... 92
Anœstheti, a new local ... 189
Anœsthetic canadol, a new local ... 109, 470
Anœsthetic powers of the Hoya poison ... 267
Anœsthetic revelation, the ... 471
Anœsthetics, a valuable lesson respecting, Dr. J. J. Chisholm ... 219
Anatomical material ... 75
Andes, the sinking cordillera of the ... 192
Ani pruritus ... 230, 233, 273
Ano, fistula in ... 180, 430
Anti emetica mistura ... 273
Antifebrin ... 102, 223, 228, 262, 338, 390, 393
Antifebrin and antipyrin ... 102, 223
Antifebrin as a nervine ... 228
Antifebrin, experience with ... 338
Antifebrin, formula for ... 393
Antipyretics ... 68, 173, 224, 273, 314, 346

Antipyrin......20, 100, 102, 173, 186, 190, 223, 224, 229, 273, 309, 346, 386, 430, 465
Antipyrin and antifebrin, relative value of......102, 223
Antipyrin and sweet spirits of nitre......346
Antipyrin as a hæmostatic......100, 186
Antipyrin as as uterine sedative......102
Antipyrin as an agalactic......430
Antipyrin, impure......309
Antipyrin in acute bronchitis in children.. 20
Antipyrin in gonorrhœa......273
Antipyrin in headaches......224
Antipyrin in labor......229
Antipyrin in nocturnal emissions......190, 386
Antipyrin in painful diseases, hypodermics of......173
Antipyrin in pertussis......465
Antiseptic action of chloroform water......380
Antiseptic, naphthol as an......305
Antiseptic, sanitos as an, Dr. F. B. Jerset....255
Antiseptic treatment of typhoid......388
Antiseptics, intestinal...... 77
Antiseptics, oxycyanide of mercury the best of......390
Aperient pill...... 35
Aphorisms, medical......105
Aphrodisiac......423
Apomorphia......184
Apparitions, warning......151
Area of dry land......272
Aristocracy, medical......316
Arkansas Medical Association......155
Arnica......110
Arsenic for warts......106
Artificial respiration......311
Asclepios syriaca for lumbago and dropsy..177
Aseptic, antiseptic and septic surgery Prof. J M F. Gaston......362
Association, American Medical......76, 158, 213, 315, 316
Association for the development of science, American......391
Association, Mississippi Valley Medical......355
Association of Arkansas, Medical...... 155
Association of Alabama, Medical......235
Association of Georgia, Medical......75, 198, 276
Association of Obstetrics and Gynecologists......315
Association, Ogeechee Medical......77, 252
Association Rhinological, American......276
Association, Southern Surgical and Gynecological......354, 394
Association, Sweet Water Medical...... 76
Asthma......70, 73, 256
Asthma, pyridin in...... 70
Asylum in Milledgeville, Inebriate......478
Atlanta Dental School......396
Atlanta Society of Medicine...... 37
Atropia on the suckling child, effects of......310
Atmospherical electricity......112
Auk, the home of the great......351
Aunts and their ways......391
Avulsions of ingrowing nails under hypnotism...... 69

B.

Bacilli of intermittent fever......264
Bacilli of typhoid, action of boiling water on......350
Bacilli of typhoid, detection of......431
Bacteriology, practical usefulness of...... 72
Badge for physicians, a...... 36
Barry, Dr. A. L......155
Bath, turkish......459
Bathers, rule for......390
Battey vs. Lawson Tait......423
Beauchamp, Dr. J. C......446
Bichloride of mercury in external eye diseases......147
Biliary calculus...... 30
Birds, wing setting of...... 72
Birth to a white and a black child at one labor giving......470
Birthday, Bobbie's......218
Bladder, inflammation of the neck of the, Dr. J. Thad Johnson......121, 166
Boil, to abort......433
Boland, Dr. K. H...... 43
Boracic acid, leucorrhœa treatment, the......382
Boracic acid, use of......429
Bock, Dr. Edward......455
Bouchelle, Dr. L. B...... 89
Bowels, obstruction of the...... 61
Boyhood, Savagery in...... 151
Brain, duality of the, Prof. R. C. Word...... 81
Britain, obstetrics and gynecology in Great, Dr. E. S McKee......147
Bromide, relative value of......350
Bromidrosis......434
Bronchitis, acute......194
Bronchitis, chronic......234, 274
Bronchitis in children, acute...... 20
Bronchitis, fetid......154
Brown, Dr. J. W...... 9
Burns and scalds......425, 465

C.

Calculus, biliary...... 30
Callus, pressure paralysis from a, Dr. Schwartz......464
Calomel......258, 418
Calomel in phagedena......258
Canadol, a new local anæsthetic......109, 470
Cancer, contagiousness of......347
Cancer treated by erysipelas inoculation, Mammary......304
Carbolic acid in hemorrhoids......53, 418
Carbolic acid in piles, Dr. F. L Haynes...... 53
Carbolic acid in typhoid fever......385
Carbuncle, treatment for......22, 425
Carcinoma uteri, treatment of......383
Cascara sagrada......21, 29, 233, 300, 339
Cascara sagrada in constipation...... 21
Cascara sagrada in rheumatism......339
Case, a remarkable......428
Case emergency......421
Cases, notes of, Dr. G. G. Roy......241
Castration in epilepsy...... 70
Castration, self Dr K. H. Boland...... 43
Cataracts, artificial ripening of......106
Catarrh, chronic...... 73
Catarrh cures......467
Catarrh, gastro-intestinal...... 35
Catarrh, nasal...... 73
Centenarian professor......195
Cerebro spinal meningitis......175
Chafing infants...... 34
Change of life and its neurosis......337
Chassaignac, Dr. Chas...... 94
Cheever, Dr. David W......458
Child, effects of atropia on the suckling..... 310
Children, acute tonsilitis in......428
Children, digestive disorders in......428
Children, dyspepsia in......234
Children, incontinence of urine in......354
Children, laxative in......478
Children, tyrotoxicon and diarrhœa of......348
Children, veratrum in pneumonia of......344
Chills, congestive...... 33
Chisholm, Dr. Julian J......219, 287
Chloranodyne......277
Chloride of Methyl, notes on the, Dr. Wm M. Trallon......291
Chloroform and ether antagonistic to cocaine......156
Chloroform, concerning......178
Chloroform, how to administer......100, 146
Chloroform in tracheotomy for croup......347
Chloroform, methylene chloride compared with...... 60
Chloroform water, antiseptic action of......380
Chlorosis, sulphur in......261

Chlorecystotomy with recovery.....221
Cholera infantum, cold water enemata in...312
Cholera infantum, vomiting in.....313
Cholera morbus of malarial fever.....154
Chrotograph.....112
Clapp, Dr. W. J.....465
Clement, Dr. L. L.....196, 315
Clergy vs. physicians.....265
Climate, influence of forests upon rainfall and.....152
Climatology and hydrology international Congress.....395
Clinical morphologies.....299
Clinical Society of London.....334
Coal, a toad in solid.....472
Coca.....140
Cocaine.....64, 75, 110, 150, 156, 268, 274, 306
Cocaine cotton.....313
Cocaine, experience in the use of.....306
Cocaine fo neuralgia.....64
Cocaine, hydrochloric acid and.....274
Cocaine in gonorrhœa.....150
Cocaine in surgery, Dr. F. Haenel.....330
Cocaine in the vomiting of pregnancy.....268
Cocaine poisoning.....63, 303
Coccyodynia and pruritus ani.....230
Codeine, sulphate of.....227
Cod liver oil, substitute for.....473
Coffee and typhoid fever.....148, 252
Cold, Dr. H. C. Woods.....354
Cold water enemata in cholera infantum...417
Collaborator, ou.....275
Collapse, introvascular injections in.....308
College, Southern Medical.....117, 235
College, The New York.....309
Collinsonia canadensis.....136, 468
Collodion salicylic.....193
Comedones.....473
Commencement Southern Medical College.117
Committees, Georgia Medical Association District.....276
Communicability of disease from animals to man.....269
Complaint, summer.....340
Congress for the study of tuberculosis.....469
Congress of hydrology and climatology, international.....396
Connecticutt, divorces in.....314
Contempt of court.....76
Constipation, cascara sagrada in.....21, 35, 353
Constipation.....35
Constipation, infantile.....27
Constipation, reflex neurosis due to.....269
Consumption, another cure for.....108
Consumption, gaseous enemata in.....65
Consumption, recent advances in treating.. 50, 65, 108
Copy man, sample.....156
Cord, treatment of unbilical.....342
Cord, tying the unbilical.....185
Corn, oil of.....350
Corpse, transplantation of skin from a..430, 435
Corrosive sublimate as a practical disinfectant.....466
Corrosive sublimate solution, how to make a.....34
Cortelyou, Dr, P. L.....196, 213, 321
Cotter, Dr. R. O.....164
Cotton seed oil, how to detect.....190
Cough in phthisis.....353
Cough, whooping.....76, 105, 173, 179, 308 474
County medical societies.....158
Cow, scarlatina and the.....76
Cramps in the leg.....186
Cranium, gunshot wounds of the.....309
Crawford, Dr. S. P.....205
Crazy? who is never.....191
Creoline.....266
Creasote in phthsis.....341
Croton oil, a large dose of.....30
Croup, chloroform in tracheotomy for.....347
Croup, prophylaxis of.....109
Cusick, Dr. W. A.....252
Cyclone, difference between a tornado and..112
Cystisin in migraine.....349
Cystitis, new remedy for.....181

### D.

Delirium tremens, methylal in.....430
Dental institution.....355
Dental matrix, a.....151
Dental School of Atlanta complimented.....396
Diabetes, a new remedy for.....421
Diaphoretic, Diuretic and sedative pill, Dr. J. B. Johnson.....336
Diarrhœa.....230 321, 348, 377, 422, 427, 457, 473
Diarrhœa, a valuable remedy for chronic...422
Diarrhœa, chronic.....427
Diarrhœa of children, lactic acid in.....230
Diarrhœa of children, tyrotoxicon and.....348
Diarrhœa of infancy and childhood, Dr. Louis Starr.....377
Diarrhœa of infants. Dr. P. L. Cortelyou.....321
Diet, good daily.....226
Digestive and laxative potion.....234
Digestive disorders in children.....428
Digitalis as a diuretic and laxative.....394
Digits, preservation of several.....464
Diphtheria,.....103, 104, 149, 176, 265, 393, 425, 426
Diphtheria carried by turkeys.....265
Diphtheria of the ear.....104
Diphtheria, some remedies in.....103
Diseases communicated from animals to men.....269
Dishonor, professional, Dr. E. van Goldtsnoven.....397
Disinfectant, antipyrin as a hœmostatic and.....186
Disinfectant, corrosive sublimate as a practical.....463
Dislocation of the shoulder, Prof. J. M. F. Gsaton.....41
District Committee, Georgia Medical association.....276
Divorces in Connecticutt.....315
Dixon, Dr. Arch.....1
Doctor's puff.....235
Dodson, Dr. N. M.....92
Dog Doctors.....156
Donhoff, Dr. E. von.....209
Drachm mark, change of.....196
Drachm grain plan of writing prescriptions Dr. C. H. Merricks.....328
Dropsy, asclepias in.....177
Drunkenness, a cure for.....11, 350
Drunkenness, quassia in.....430
Duality of the brain, Prof. R. C. Word.....81
Dublin obstetrics, Dr. E. S. McKee.....324
Dubois, Dr. Henry A.....372
Dysentery.....32, 68, 233, 275, 385, 394
Dysentery in Texas.....68
Dysentery, naphtholine in.....394
Dysmenorrhœa.....269, 393
Dysmenorrhœa, formula for.....393
Dysmenorrhœa treated by mental suggestion or hypnotism, Dr. A. C. Hugenschmidt.....453
Dyspepsia.....234, 343, 426, 353
Dyspepsia, hydrochloric acid in.....234
Dyspepsia in children.....234
Dyspepsia, strychnine in.....426
Dyspepsia with chronic constipation and sour stomach.....353

### E.

Ear, diphtheria of the.....104
Ear, inflammation of the middle, Dr. R. O. Cotter.....164
Earache.....433
Earth at the French exposition, model of..471
Eclampsia puerperal, Dr. F. M. Rushing.....326

Eclectic remedies....325
Eczema.... 74, 434
Eczema of the head....434
Edison's phonograph.... 72
Edison's photophone....272
Elastic mucilage, a new....270
Electric shaft, a visit to the....157
Electricity as a pain killer.... 25
Electricity, atmospherica.... 112
Electricity in post partum hemorrhage.... 56
Electrolysis in stricture, Dr. J. J. Berry....250
Embalming fluid....187
Emergency case....421
Emetic, nitrate of potash as an.... 28
Emissions, antipyrine in nocturnal....190, 386
Emulating perfection....317
Enemata, glycer nein....110, 469
Enemata syringe, the.... 57
Engineering invention....432
Enterotomy, four cases of, Dr. E. von Donhoff....209
Ephedrine, a new myebriatic....107
Epilepsy....70, 474
Epilepsy, castration in.... 70
Epistaxis and hemorrhage, Dr. B. Frank Humphries....410
Epistaxis, uncontrollable, Dr. J. J. Chisholm....287
Epithelioma, remedy for....188
Ergot and acetic acid in post partum hemorrhage....145
Erysipelas inoculation, mammary cancer treated by....304
Erythropleine....188
Esmark, Dr. von....435
Ether and chloroform antagonistic to cocaine....156
Explosive, a new flameless....232
Exposition, model of the earth at the French....471
Eye, bichloride of mercury in external diseases of the....147
Eye, photographs of the....432
Eye, removing particles from the....109

### F.

Facial neuraligia, salicylate of sodium in...110
Fat, how to grow.... 84
Female physicians in New York City....156
Fever enteric....141
Fever, intermittent....157, 264
Fever intermittent, baccilli of....264
Fever malarial....154
Fever typhoid....74, 113, 114, 148, 182, 218, 229, 252, 354, 385, 388, 417
Fever, yellow....381, 389, 391
Fire and water proof paper....391, 431
Fire, causes of....471
Fistula in ano....180, 430
Fistula, a large vesico-vaginal, Dr. K. P. Moore....405
Flannel underwear, poisoned by....107
Flies, to protect horses and cows from....110
Fluid, embalming....187
Forceps, spoonhandle, Dr. J. Stainback Wilson....245
Forest upon rainfall and climate, influence of....152
Freire, Dr. Domingos....356
Furey, Dr. G. W.... 12
Furuncles, to abort....433

### G.

Gallstones passed by a child two and a half years old, forty-three....461
Gallstones, treatment of.... 346, 461
Galvanism for indolent ulcers.... 64
Gann, Dr. Dewell....196, 235
Gas, a new....71, 152
Gaston, Prof. J. M. F....41, 201, 282, 356, 362, 396
Gastric juice, relations between salvia and..182
Gastric tonic, a laxative....194
Gastro-intestinal catarrh.... 35
Gelseminum for rigid os....195
Georgia, Medical Association of...75, 155, 198, 276
Geranium macu atum in chronic bronchitis.... 234
Germany, obstetrics and gynecology in, Dr. E. S. McKee.... 45
Geyser, he grea yellowstone....272
Gila monster, venom of the rattlesnake and the....468
Gleditschine....267
Gleet....113
Glycerine in enema a....10, 469
Glycerine in habitual constipation....389
Goldtsnoven Dr. E. van....397
Goitre....39, 189, 349, 421, 422
Goitre can be cured....349
Goldsmith, death of Dr. Middleton.... 75
Gonorrhœa....25, 113, 127, 150, 273, 422
Gonorrhœa, hints in the treatment of.... 25
Gray, Dr. L. C....447
Greenly, Dr. T. B.... 414
Growths of the nasal cav ities, Dr. A. G. Hobbs....401
Guacal....104
Guaiac in acute tonsilitis.... 70
Gunshot wounds of the cranium....309
Gynecological Association, Southern Surgical and....355, 395
Gynecologists, American Association of Obstetricians and....315
Gynecology in Germany, Dr. E. S. McKee... 45
Gynecology in Great Britain Dr. E. S. McKee....247

### H.

Haenel, Dr. F....330
Hair tonic....274
Hardman, death of Dr.... 75
Hasheesh, psychic effects of....431
Hawthorn in uterine hemorrhage, red....188
Haya poison, its anæsthetic powers....267
Haynes, Dr. Francis L.... 53
He comes back....436
Head, eczema of the....434
Head, neuralgia of the....141
Headache....393, 394, 467
Headache a table of 39 kinds of....467
Heart, a sympathetic....352
Heat fever or sunstroke....298
Hemorrhage, epistaxis and, Dr. B. Frank Humphries....410
Hemorrhage internal....354
Hemorrhage of the lungs....354
Hemorrhage of the palm of the hand....423
Hemorrhoge, post partum....56, 145, 189
Hemorrhage, uterine....99, 109, 265
Hemorrhoids....274
Hemorrhoids, injection for....24, 67
Hemorrhoids, novel treatment of....148
Hemostatic, antipyrine as a....100
Hepatic congestion....315
Herring, Dr N. B....215
Hip dislocation....190
Hoagland laboratory, the....475
Hobbs, Dr. A. G....401, 435
Home journals, support your.... 75
Hooping cough....76, 105, 113, 179, 308, 474
Horses have sense? do.... 71
Hospital, Ivy street....354, 397
Hot air inhalations for phthisis....462
How to grow fat....184
Hudson river tunnel....432
Hugenschmidt, Dr. Arthur C....453
Humbug, a new foreign....236
Humphries, Dr. B. Frank....410
Hydrastis canadensis in uterine hemorrhage.... 99

Hydrocele....183, 388
Hydrochloric acid and cocaine....274
Hydrochloric acid in dyspepsia....234
Hydrology, international congress of climatology and....395
Hydrophobia....115, 186
Hydrophobic tetanus, Dr. J. J. Chisholm....289
Hydrotherapy during menstruation....427
Hypnotic for alcoholism, a....313
Hypnotism....172, 453, 475
Hypnotism, dysmenorrhœa treated by, Dr. A. C. Hugenschmidt....453
Hypnotism in therapeutics....172

### I.

Ice on Mars....352
Imagination, man's power of....310
Impotence, functional....74
Incendiary, the sun as an....272
Incompatibility, an important....190, 235
Incontinence of urine in children....354
Indian hemp, treatment of migraine with....345
Indigestion acid....307
Inebriate asylum in Milledgeville....478
Infancy and childhood, diarrhœa of, Dr. L. Starr....3 7
Infant food, kephir as an....308
Infants, chafing....34
Infants, diarrhœa of, Dr. P. L. Cortelyou....321
Ingrowing toe nail....69, 189
Ingrowing nail under hypnotism, avulsion of....69
Injection in collapse, introvascular....308
Ink, removing indelible....394
Inoculation against yellow fever....235
Insects stings....193
Insomnia....33
Institution, a noble....397
Instruction, dental....355
Intercranial neuralgia....268
International congress of hydrology and climatology....395
Intestinal affections....77, 185
Intestinal diseases of children....185
Intestinal obstruction....61, 63, 149, 260
Intubation....102, 259, 345
Intubation of larynx....259
Intubation or tracheotomy....102
Inventions, engineering....432
Iodide of ammonia in asthma....256
Iodine in vomiting during labor....150
Iodoform, a substitute for....87
Iodoform and tar....470
Iodoform poisoning....427
Ipecacuanha, Dr. L. B. Bouchelle....89
Iron blue, to color....432
Iron, how to give....218
Iron, wrought....152
Irregularities, menstrual....26, 420
Itch, simple treatment of....353
Ivy street hospital....354, 397

### J.

Jaborandi in obstetric practice....266
Jenks' memorial prize, the William F....115
Jesset, Dr F. B....255
Johnson, Dr. J. B....336
Johnson, Dr. Jno. Thad....121, 166, 235
Journal, don't stop your....475
Journal, our....475
Journal, write for your....436
Journals, support your home....75

### K.

Kephir as an infant food....308

### L.

Labor, antipyrine in....229
Labor, tincture of iodine in persistent vomiting during....150
Laboratory, the Hoagland....435
Lactic acid in diarrhœa of children....230
Lallerstedt, Dr. T. L....155, 315
Lane, Dr. R. Y....252
Laparatomy in peritonitis....106
Laparatomy, intestinal obstruction relieved by....260
Laparatomy in volvulus, Prof. J. F. M. Gaston....201
Laryngoscope, Leiter s....152
Larynx, intubation of the....259
Lawless liberty....476
Lawson, Dr. H. M....172
Laxative, digitalis as a diuretic and....394
Laxative gastric tonic, a....194
Laxative in children....478
Laxative potion, a digestive and....234
Leg, how to treat cramps in the....186
Leg ulcers....187
Leprosy through vaccination....152
Leucorrhœa....434, 382
Leucorrhœa treatment, the boracic acid....382
Ligatures, how to preserve....143
Light from the stars....192
Lightning conductors, Prof. Tyndall on....31
Lima, Peru sanitary conference i....75
Lime, phosphate of....30
Liniment, good samaritan....34
Lipanine....466
Liquids by pressure, solidification of....112
Little things in medicine, Dr. N. B. Herring....215
Liver, suture of a wound of the....470
London Clinical Society....334
Louisiana State Medical Society....236
Lugol's solution....354
Lumbago, asclepias in....177
Lungs, hemorrhage of the....354

### M.

Mackenzie's book, Dr....437
McKee, Dr, E. S....45, 247, 324
Magazine guns....271
Magnet, detention of foreign bodies with the....109
Malaria, chronic....34
Malarial attacks, hypodermics in....394
Maldonite, an Australian mineral....151
Mammary cancer treated by erysipelas inoculation....304
Man an object of zoology, Dr. S. W. Stiles....3
Man, the musquito a blessing to....352
Man's power of imagination....310
Mars, ice on....352
Martin, Dr. Robt. W....16
Mastitis treatment of....263
Matrix, a dental....151
Medical and Surgical Reporter, another....396
Medical aristocracy....316
Medical Association, Alabama....235
Medical Association, American....76, 158, 213, 315, 316
Medical Association, Mississippi Valley....354
Medical Association of Arkansas....155
Medical Association of Georgia....75, 198, 276
Medical Association of Ogeechee....77, 252
Medical Association of Sweet Water....76
Medical College, Southern....117, 235
Medical progress, monthly summary of....463
Medical societies, county....158
Medical Society, Louisiana State....236
Medicine, Atlanta Society of....37
Medicine, little things in, Dr. N. B. Herring....215
Medicine, modern thoughts in, Prof. T. S. Powell....131
Meningitis, cerebro-spinal....175, 353
Meningitis in children, treatment of....353
Menstrual irregularities, a valuable formula in.....26, 420
Menstruation, hydrotherapy during.....427
Menstruation, opium habit and.....387
Menthol and mentholeata.....189
Mercury bichloride in external disease of the eye.....147
Mercury oxcyanide, the best of antiseptics..390
Mercury salicylate in syphillis.....261
Merrick, Dr. C. M.....328
Meteorites' origin of.....232
Methyl refrigerating by chloride of.....228
Methyl in delirium tremens.....430
Methylene chloride compared with chloroform.....60
Microbe, yellow fever.....355
Migraine.....268, 345, 349
Migraine, cystisin in.....349
Migraine with Indian hemp, treatment of..345
Milledgeville, inebriate asylum in.....478
Miller, Dr W.....399
Mind cure, Dr. S P. Crawford.....205
Mississippi Valley Medical Association.....355
Mistura acidi muriatici.....273
Mistura anti emetic.....273
Mistura rhei et calcis.....273
Mistura, muriate of ammonia compound...273
Monitors.....432
Moore Dr. K. P.....277, 405
Morphine addiction, the curability of.....295
Morphio-mania, the treatment of..... 28
Morphine poisoning, tracheotomy in.....257
Morphologies, clinical.....299
Musquito a blessing to man, the.....352
Mouth wash, a..... 194
Mucilage, a new elastic.....270
Mullein oil in enuresis.....269
Muriate of ammonia, compound mixture...273
Myalgia, muriate of ammonia i.....433
Mydriatic, ephedrine a new.....107
Myxœdema.....334

N.

Nævi, painless destruction of.....69, 429
Nail, ingrowing.....69, 189
Nail under hypnotism, avulsion of ingrowing..... 69
Naphthaline, in dysentery.....394
Napthol as an antiseptic.....305
Nasal cavities, gummatous growths of the, Dr. A. G. Hobbs.....401
Neonatorum ophthalmia.....388
Nephrotomy, Dr. Arch Dixon..... 1
Nervine, antifebrin as a.....228
Nervous headache.....393
Nesbit's address, Judge.....126, 155
Neuralgia.....64, 68, 110, 141, 269, 433, 447, 474
Neuralgia, a remedy for.....110
Neuralgia, cocaine in..... 64
Neuralgia, facial.....110
Neuralgia, head.....141
Neuralgia, intercranial..... 68
Neuralgia, sulphur in sciatic.....424
Neuralgia, modern treatment of, Dr. L. C. Gray.....447
Neuralgia, pomade for.....474
Neurosis due to constipation, reflex.....269
Night sweats, a new remedy for.....148
Nipples, sore.....66, 274, 313
Nitre, antipyrine and sweet spirit of.....346
Nitroglycerine, indications for the use of..... 23
Nose, source of all our woes, the.....111
Notes of cases, Dr. G. G. Roy.....241
Nursing women upon their infants, influence of medicine taken by.....263
Nux vomica in different individuals, peculiarities of, Dr. Chas. Chassaignac..... 94

O.

Oak, a gigantic.....311
Obesity, German treatment of.....460
Obstetric anæsthesia, Dr. N. M. Dodson..... 92
Obstetric practice, jaborandi in.....266
Obstetricians and Gynecologists, American Association.....315
Obstetrics, Dublin, Dr. E. S. McKee.....321
Obstetrics, Germany, Dr. E S. McKee..... 45
Obstetrics in Great Britain.....247
Obstructions, intestinal.....61, 63, 149, 260
Obstruction of the bowels, operative treatment of..... 61
Ogeechee Medical Association.....77, 252
Oil, a substitute for cod liver.....473
Oil of corn.....350
Oil of mullein in enuresis.....269
Ointment, pile.....273
Ophthalmia neonatorum.....388
Ophthalmic surgery of the present century 18
Opium habit and menstruation.....387
Opium poisoning cured by amyl netrite.....180
Ordnance that has ever been fired, what is the longest.....472
Os, gelseminum for rigid.....195
Ostriches.....391
Otitis.....310
Ovaries vs. testicles, Dr. Edward orcs.....455
Oxcyanide of mercury the best of antiseptics.....390

P.

Pain killer, electricity as a..... 25
Pain, obtunder after extraction.....194
Palmer, death of Dr. A. B..... 75
Paralysis from a callus, pressure, Dr. Schwartz.....464
Paraphymosis, reduction of.....430
Paris Exposition, American Exhibit at the.436
Paris Exposition, Model of the Earth at the.472
Parke, Dr. A. T.....196
Pathogenesis of pterygium..... 21
Pearl upon the rose, Dr. Henry A. Dubois...372
Peppermint, oil of.....235
Peppermint water in pruritus pudendi.....304
Perfection, emulating.....317
Perinephritic abscess, Dr. G. G. Roy.....242
Peritonitis, acute..... 69
Peritonitis, laparatomy in.....166
Pertussis, antipyrin in.....465
Petition by a native East Indian.....108
Petroleum, the origin of.....231
Phagedena, calomel in.....258
Pharyngitis, follicular..... 73
Phonograph, Edison's..... 72
Phosphate of lime..... 30
Photograph of the eye.....432
Photophone, Edison's.....272
Photophoxyline.....387
Phthisis.....27, 194, 341, 353, 462
Phthisis, agaricin in.....194
Phthisis, cough in.....353
Phthisis, creasote in.....341
Phthisis, hot air inhalation for.....462
Phthisis, hypodermis of sodium phosphate in..... 27
Physicians, a badge for..... 36
Physicians, a plea for a more amicable relationship between, Dr. T. B. Greenley.....414
Physicians in New York city, female.....156
Physicians, the clergy versus.....268
Piffard, Dr. H. G..... 96
Pil ointment.....233
Piles internal.....273
Piles treated by carbolic acid, Dr. Francis L Haynes..... 53
Pill aperient..... 35
Pill, diuretic, sedative and diaphoretic, Dr. J. B. Johnson.....336
Pilocarpine in diptheria.....426
Placenta prævia.....178, 244
Pleurisy.....474
Pleuritis.....125, 142

Pneumonia......61, 107, 344, 429, 474
Pneumonia, hot pack in......419
Pneumonia of children, veratrum in......344
Pneumonia, venesection in...... 62
Poisoned by flannel underwe r...... 107
Poisoning...... 63, 107, 180, 303, 413, 427
Poisoning, a case of opium......180
Poisoning, iodoform......427
Poisoning, rhus...... 413
Polyuria......154
Pomade for neuralgia......474
Potash as an emetic, nitrate of...... 28
Potassium permanganate in tooth ache......189
Potion, a digestive and laxative......234
Powell, Dr. T. S......181, 155, 476
Pregnancy, chloride of sodium in......298
Pregnancy, uterine hemorrhage of......265
Prescriptions, dram-grain method of writing, Dr. C. M. Merrick......328
Prize, the William F. Jenks memorial......115
Professional dishonor, Dr. E. van Goidtsnoven......397
Professors, centenarian......195
Pruritus ani......230, 233, 273
Pruritus pudendi, peppermint water in......304
Psychie, effects of hasheesh......431
Pterygium, the pathogenesis of...... 21
Puerperal eclampsia, Dr. F M. [illegible]ushing......326
Puffs, doctors'......235
Puncture, treatment of tympanitic......297
Pupil as a guide in the administration of chloroform......146
Purgative mixture......193
Pyæmia...... 113
Pyridine in asthma...... 70

Q.

Quack advertisements in religious newspapers......115
Quassia in drunkenness......430
Quinine in gonorrhœa......147

R.

Rabies, chloral hydrate in......109
Rabies from Tanacetic acid...... 67
Rainfall and climate, influence of forests upon......152
Rape as defined by the Texas Supr'e Court 67
Rattlesnakes, gila monster, venom of the...468
Reflex neurosis, due to constipation......269
Refrigeration by chloride of methyl, local..228
Relationship between physicians, a plea for better, Dr. T. B. Greenley......414
Religious newspapers, quack advertisements in......116
Remedies, Eclectic......235
Respiration, simple method of artificial......311
Revaccination, the age for......204
Revelation, the anæsthetic......471
Rhei et calcis mistura......273
Rheumatism......65, 108, 113, 190, 270, 339, 434
Rheumatism, cascara sagrada in......339
Rheumatism in the Jefferson College Hos.. 65
Rheumatism, salol in......190
Rheumatism, theine in......108
Rhinological Association, American......276
Rhus poisoning......413
Ring worm......353
Rose, the pearl upon the, Dr H. A. Dubois..372
Roy, Dr. G. G., notes of cases......241
Rushing, Dr. F. M......326

S.

Saccharin......146, 384
Salicylate of sodium......105, 1 0, 184, 267
Salicylate of sodium in facial neuralgia......110
Salicylate of sodium in tonsilitis......267
Salicylate of sodium in whooping cough......105
Salicylate collodion......193
Salicylic plaster, compound...... 74
Saliva and gastric juice, relation between....182
Salivation, chronic syphilitic......429
Salol in rheumatism......190
Salt in hygiene and on skin. Dr. H. G. Piffard...... 96
Samaritan liniment, good...... 34
Sample copy man......156
Sanitary conference in Lima, Peru...... 75
Sanitas as an antiseptic, Dr. F. B. Jessel......255
Savagery in boyhood......151
Saw, invention of circular......432
Scarlatina and the cow...... 76
Scalds, burns and......425
Schwartz of Paris, Dr......461
Sciatic neuralgia, sulphur in......424
Sciatic, treatment of......9, 29, 140, 218, 424
Sciatic, three cases of, Dr. J. W. Brown...... 9
Science, American society for the development of......391
Science, the growth of......271
Sea telephone, a...... 31
Sedative, diuretic and diaphoretic pill, Dr. J. B. Johnson......236
Sensible...... 30
Sexes, law of proportion of......108
Sextuple......347
Shock, Dr. D. W. Cheever......458
Shoulder, dislcoation of the, Prof. J. M. F. Gaston...... 41
Shoulder dislocation, rapid reducing of......188
Shoulder presentation, a case of, Dr. J. C. Beauchamp......146
Silk, artificial...... 71
Skin from a corpse, transplantation of..430, 435
Skin, new method of treating diseases of the...... 26
Skin, salt in treatment of the, Dr. H. G. Piffard...... 96
Sleeplessness, treatment of......222
Snake bites and yellow fever......391
Snakes, self mending......354
Societies, county medical......158
Society, London Clinical......334
Society of Medicine, Atlanta...... 37
Sodium chloride in pregnancy......298
Sodium, iodide of...... 29
Solidification of liquid by pressure......112
Solution, Lugol's......354
Soot......110
Sparteine, sulphate of......104
Spearman, Dr. G. C......161, 196
Sponges, cultivation of......311
Stars, light from the......192
Stars, remote......192
Starr, Dr. Louis......377
Stenocarpine......267
Sterility, one child......386
Steynberg, Dr. George M......356, 389
Stiles, Dr. S. W...... 3
Stings, insect......193
Stomach, dyspepsia with sour......353
Stomatitis...... 73
Stones from a child two and a half years old, forty-three gall......461
Stricture, electrolysis in, Dr. J. J. Berry......250
Strophanthus......59, 465
Strophanthus, tetanus treated by, Dr. W. J. Clapp......465
Strychnia in acoholism......309
Strychnia in dyspepsia......426
Substitution......197
Suckling child, effects of atropia on the......310
Sulphonal......344
Sulphur in chlorosis......261
Summary of medical progress, monthly......463
Sun as and incendiary, the......273
Sunstroke......298
Surgery, abdominal......307
Surgery, cocaine in......330

Surgery of the present century, ophthalmic 18
Surgical and Gynecological Association, Southern.....355, 395
Suture of a wound.....470
Sympathetic heart, a.....352
Syphillis, chronic, Dr. H. M. Lawson.....172
Syphillis in the early stages.....154
Syphillis, salicylate of mercury in.....261
Syringe, the enemata..... 57

T.

Tait, Båttey vs. Lawson.....423
Talc, chronic diarrhœa treated by.....427
Tampon, a vaginal.....429
Tampon, how to.....225
Tanacetic acid, rabies from..... 67
Tangent in the world, the longest.....472
Tape worms, new treatment for.....154
Tar.....150, 470
Tar, iodoform and.....470
Tea, effects of.....150
Teeth, changes in..... 72
Telegraphy, long distance.....211
Telephone, a sea..... 31
Temperature as a symptom, Dr. Robert A. Martin..... 16
Testicles vs. ovaries, Dr. Edward Borck.....455
Tetanus.....289, 465, 474
Tetanus hydrophobia, Dr. J. J. Chisholm.....289
Tetanus treated by strophanthus, Dr. W. J. Clapp.....465
Texas Health Journal.....236
Texas, treatment of dysentery in..... 68
Texas Supreme Court, rape as defined by the 67
Thallon, Dr. William M .....291
Theine in rheumatism.....108
Toad in solid coal, a.....472
Therapeutics, hypnotism in.....172
Think, the time in which we.....231
Things in medicine, little, Dr. N. B Heriing 215
Thymus vulgaris in whooping cough.....308
Tonic, a laxative gastric.....194
Tonic, hair.....274
Tonsilitis.....25, 70, 113, 267, 310, 393 428, 436
Tonsilitis, acute..... 70, 393
Tonsilitis, guaiac in.....70, 113, 428
Tonsilitis in children, acute.....428
Tonsilitis treated by sodium salicylate.....267
Tonsil, enlarged..... 25
Tooth, pain obtunder after extraction of a..194
Tooth without pain, to extrac a..... 28
Toothache' potassium permanganate in.....189
Toothache, to stop a .....313
Tornado, difference between a cyclone and a.....112
Touch, the finishing.....107
Tracheotomy for croup, chloroform in.. .....347
Tracheotomy in morphine poisoning.....257
Tracheotomy, intubation or.....102
Trichiniasis, Dr. G. W. Furey..... 13
Tribrimphenol.....226
Tuberculosis, a cure of .....306
Tuberculosis, report of the delegate to the Congress for the study of.....469
Tunnel, Hudson River.....432
Turkeys, diphtheria carried by.....265
Tuto cito et jucundi .....116
Twins, one white and one black at one labor.....352
Tympanitis by puncture, treatment of.....297
Tyndall'on lightning conductors.....31
Typhoid, action of boiling water on bacilli of.....350
Typhoid, antiseptic treatment of.....388
Typhoid baccilli, detection of.....431
Tyyhoid fever.....74, 113, 114, 148, 182, 218, 229, 252, 354, 385, 388, 417
Typhoid fever, aborting.....182
Typhoid fever, carbolic acid in.....385
Typhoid fever, coffee in, Dr. W. A. Cusick..252
Typhoid fever, delirium of.....74, 113, 114, 218
Typhoid fever in children.....226
Tyrotoxicon, Dr. W. C. Vaughan.....5, 348
Tyrotoxicon and diarrhœa of children.....348

U.

Ulcers, indolent.....19, 64, 74, 187, 469
Umbilical cord, treatment of.....342
Umbilical cord, tying the.....185
Unconscious persons, simple methods for reviving.....191
Uric acid calculi, biborate of ammonia in. .139
Urine in children, incontinence of.....354
Urine in women, incontinence of.. .....426
Uteri, treatment of carinoma.....383
Uterine hemorrhage.....99, 109, 188, 265
Uterine hemorrhage, hydrastic canadensis in.....99, 265
Uterine sedative, antipyrine as a.....102

V.

Vaccination, leprosy through.....152
Vaginal tampon, a.....429
Vaughan, Dr V. C..... 5
Venom of rattlesnakes and the gila monster.....468
Venesection in pneumonia..... 62
Veratrum viride.....23, 310, 326, 341
Veratrum viride, a specific in puerperal eclamysia, Dr. F. M. Rushing.....326
Veratrum viride in pneumonia of children.344
Vesico-vaginal fistula, a large, Dr K. P. Moore.....405
Volvulus, laparatomy in, Prof. J. M. F. Gaston.....201
Vomiting and its treatment, Prof. R. C. Word.....442
Vomiting during labor, iodine in.....150
Vomiting in cholera infantum.....313
Vomiting, paroldehyde in obstinate.....307

W.

War ships and forts, great.....111

Wartcharming, extraordinary.....270

Warts treated internally by arsenic.....106

Wash, a mouth.....194

Water proof paper, fire and ..... 391, 431

What is the matter with me? Dr. G. C. Spearman.....161

Whitty, Dr. T. R..... 76

Who is never crazy?.....191

Whooping cough.....76, 105, 113, 179, 308' 474

Why is it?.....235

Wine, how made now a days..... 70

Wings, setting of birds..... 72

Women, incontinence of urine in.....426

Word, Prof. R. C.....81, 442, 476.

Worm, ring.....353

Worms, .....434

Wounds of the cranium, gunshot.....309

Wrinkles.....256

Write for your journal.....436

Wrought iron, expansion and contraction...152

Y.

Yellow fever.....381, 389

Yellow fever, inoculation against.....235, 399

Yellow fever, inoculation against, Dr. W. Miller.....339

Yellow fever microbe.....355

Yellow fever, pathogenesis of.....110

Yellow fever, snake bite and.....391

Yellowstone geyser, the great.....272

RECEIPTED.—1887—Drs. T. A. Gibson, W. S. Glass, A. V. Ponder, A. M. Duncan, J. B. Price, Richard Inge, W. S. Johnson, W. B. Maxwell, R. B. Stapleton, to July; A. B. Loving, Jno. W. Sanders.

1888.—W. W. Leak, T. L. Appleby, J. M. Bolger, H. L. Sutherland, James Roach, Tho. A. Birch.

